# Improving health workforce governance: the role of multi-stakeholder coordination mechanisms and human resources for health units in ministries of health

**DOI:** 10.1186/s12960-022-00742-z

**Published:** 2022-05-26

**Authors:** Tim Martineau, Kim Ozano, Joanna Raven, Wesam Mansour, Fiona Bay, Dominic Nkhoma, Elsheikh Badr, Sushil Baral, Shophika Regmi, Margaret Caffrey

**Affiliations:** 1 Department of International Public Health, Liverpool School of Tropical Medicine, Pembroke Place, Liverpool, L3 5QA UK; 2 Friends of Waldorf Education, Stuttgart, Germany; 3 Kamuzu University of Health Sciences, Blantyre, Malawi; 4 Arab Board of Health Specializations, Amman, Jordan; 5 HERD International, Kathmandu, Nepal

**Keywords:** Human resources for health (HRH), HRH governance, Health workforce, Coordination mechanisms, HRH unit

## Abstract

**Background:**

A cohesive and strategic governance approach is needed to improve the health workforce (HW). To achieve this, the WHO Global Strategy on Human Resources for Health (HRH) promotes mechanisms to coordinate HRH stakeholders, HRH structures and capacity within the health sector to support the development and implementation of a comprehensive HW agenda and regular reporting through WHO’s National Health Workforce Accounts (NHWA).

**Methods:**

Using an adapted HRH governance framework for guidance and analysis, we explored the existence and operation of HRH coordination mechanisms and HRH structures in Malawi, Nepal, Sudan and additionally from a global perspective through 28 key informant interviews and a review of 165 documents.

**Results:**

A unified approach is needed for the coordination of stakeholders who support the timely development and oversight of an appropriate costed HRH strategy subsequently implemented and monitored by an HRH unit. Multiple HRH stakeholder coordination mechanisms co-exist, but the broader, embedded mechanisms seemed more likely to support and sustain a comprehensive intersectoral HW agenda. Including all stakeholders is challenging and the private sector and civil society were noted for their absence. The credibility of coordination mechanisms increases participation. Factors contributing to credibility included: high-level leadership, organisational support and the generation and availability of timely HRH data and clear ownership by the ministry of health.

HRH units were identified in two study countries and were reported to exist in many countries, but were not necessarily functional. There is a lack of specialist knowledge needed for the planning and management of the HW amongst staff in HRH units or equivalent structures, coupled with high turnover in many countries. Donor support has helped with provision of technical expertise and HRH data systems, though the benefits may not be sustained.

**Conclusion:**

While is it important to monitor the existence of HRH coordination mechanisms and HRH structure through the NHWA, improved ‘health workforce literacy’ for both stakeholders and operational HRH staff and a deeper understanding of the operation of these functions is needed to strengthen their contribution to HW governance and ultimately, wider health goals.

**Supplementary Information:**

The online version contains supplementary material available at 10.1186/s12960-022-00742-z.

## Background

Tackling key systemic health workforce (HW) issues (quantitative shortage, skills-mix, distribution imbalance, and more) requires a cohesive and strategic governance approach centred around coordinating and implementing policies to gain improvements in workforce performance [1]. From 2006, the Global Health Workforce Alliance (GHWA) and WHO provided catalytic support to national Human Resources for Health (HRH) coordination mechanisms to ensure integrated actions [2]. In 2016, the World Health Assembly adopted the WHO Global Strategy on Human Resources for Health: Workforce 2030 [3], which established periodic reporting requirements for Member States, facilitated by WHO. Objective 3 of this Global Strategy on HRH (Build the capacity of institutions at subnational, national, regional and global levels for effective public policy stewardship leadership and governance of actions on HRH) has two milestones that relate to countries having “inclusive institutional mechanisms in place to coordinate an intersectoral health workforce agenda” (Milestone 3.1) and having HRH Units or departments “with responsibility to develop and monitor policies and plans” (Milestone 3.2)—see Table 1. The National Health Workforce Accounts (NHWA) [4] monitor the availability of these mechanisms and structures. However, a deeper understanding is needed of the governance, functions, attributes and performance of HRH stakeholder coordination mechanisms and HRH Units in their ability to support the “implementation of a comprehensive HW agenda in countries” ([3], para55).

**Table 1:** Definitions of HRH governance mechanisms based on the global strategy and NHWA [3, 4]

Term	Definition
HRH coordination mechanism	Institutional mechanism which includes all key stakeholders and coordinates an intersectoral HW agenda. Mechanisms may be a national coordination committee involving, for example, inter-ministerial Sustainable Development Goals committees, sector skills councils or similar high-level bodies with a leadership function for coordinating, developing and monitoring policies and plans on HW, and negotiating intersectoral relationships with other line ministries, government agencies and other stakeholders
HRH unit or department	An organisational structure reporting to a senior level within the Ministry of Health (Director General or Permanent Secretary) with the capacity, responsibility, financing and accountability for core functions of HRH policy, planning and governance, data management and reporting

### Health workforce (HW) development and governance

HW development is a technical process requiring expertise in planning, education and management, and the capacity for strategic HRH long-term planning “rooted in a long-term vision for the health system” ([3], para55). It is also a political process “requiring the will and the capacity to coordinate efforts on the part of different sectors and constituencies in society and different levels of government” ([5], p798). Governance includes rules (both formal and informal), roles and responsibilities for collective action and decision-making in a system with diverse stakeholders [6, 7]. Key challenges to intersectoral governance and coordination efforts among stakeholders are willingness, commitment, technical capacity, financial mobilisation (often in a fiscally constrained space), operational accountability and effective leadership [3].

### Stakeholders in HW development and governance

A large number of stakeholders with differing—and sometimes opposing—interests occupy the ‘HRH policy space’ [8] which all require coordinating for HW development; see Table 2 ([9], p115).

**Table 2:** Stakeholders impacting on HRH policy

Stakeholder group	Institutions
Government	Health (national, regional, local government) Executive leadership (president, prime minister, cabinet) Legislative bodies Finance Education Labour Defence and military Civil service agencies and commissions Statutory professional councils
Employers	Private for-profit businesses Public–private partnership Voluntary or non-profit-making organisations
Representatives of health workers	Professional and occupational associations Professional and occupational unions
International stakeholders	Bilateral and multi-lateral agencies Philanthropic organisations Professional and occupational organisations
Civil society	Community based organisations Patients’ rights organisations
Other stakeholders	Media Pharmaceutical and medical device companies

There is a distinct gap in the literature surrounding both the concept and function of health workforce governance across different contexts in terms of HRH coordination mechanisms and HRH units or departments. Two studies based on surveys in Africa [10] and South East Asia [11] provide information on HRH units, but lack detail on how they operate. Cometto et al. call for more understanding of the political economy of HRH policy-making and “the characteristics and performance factors, gained through the specific lens and focus on HRH units” ([11], p2). In their discussion of the Guinean HW, de Pas et al. suggest that the health workforce development should be aligned with fiscal and budgetary space to improve health sector and HW investment [12]. Therefore, the focus of this paper is on the functions, attributes and performance of the coordination mechanisms and HRH units. This is based on a study to review the situation globally and within three countries: Sudan, Nepal and Malawi.

## Methods

### Theoretical framing of the study

The Barbazza and Tello framework [13], adapted by Lim and Lin [1] for review of HRH governance, provides a comprehensive framework for reviewing coordination mechanisms and HRH units. In this study, we have adapted and expanded the Barbazza and Tello framework and have added the dimensions of ‘ownership’ from Buse and Walt [14] and ‘HRH literacy’ from Martiniuk [15] in Table 3.

**Table 3:** Dimensions of HRH governance framework for reviewing the HRH coordination mechanisms and HRH units

Dimensions of governance
Accountability
Leadership
Partnership
Ownership
Formulating policy/strategic direction
Generating information/intelligence
Organisational adequacy/system design
Participation and consensus
Sustainability
HRH performance
HRH literacy

### Study design

We used a rapid qualitative study design to address the research aim. We employed two methods: document review and in-depth interviews with a small number of selected informants (both global and specific to each of the three countries). Both methods were guided by the HRH governance framework described above.

### Study sites

Three countries, namely Malawi, Nepal and Sudan, part of the WHO regions of AFRO, SEARO and EMRO, respectively, were selected for the study. These countries signed up to the Kampala Declaration and Agenda for Global Action in 2008 commitments [16] and were supported by the Global Health Workforce Alliance programme [2]. They are on the WHO’s health workforce support and safeguard list because of staff shortages [17], and HRH functions are partially decentralised. The research team has existing links with these countries which enabled rapid data collection. Table 4 provides information about each country.

**Table 4:** Country contexts

	Malawi	Nepal	Sudan
Population	18.63 million (2019)^a^	28.60 million (2019)^a^	42.81 million (2019)^a^
Total Health workforce^d^	37,926^a^	54,177^a^	150,000^a^
Per 10,000 population			
Doctors	0.36 (2018)^a^	8.09 (2010)^a^	2.62 (2017)^a^
Nursing Personnel	4.39 (2018)^a^	21.25 (2019)^a^	11.45 (2018)^a^
Midwifery Personnel	0.33 (2016)^a^	N/A	N/A
Dentists	0.02 (2018)^a^	1.12 (2019)^a^	2.09 (2015)^a^
Pharmacists	0.06 (2018)^a^	1.35 (2019)^a^	0.25 (2017)^a^
Total stock of above cadres	1349 (2018)	63,944 (2019)	35,964 (2017)^a^
HRH strategy	2012–2016 2018–2022 (in use)	2012–2016 2020–2030 (approved)	2012–2016 2018 strategy (in progress)
Health service providers	Ministry of Health (MoH)—69%^b^ Christian Health Association of Malawi—29%^b^ Private sector providers—2%^b^	Ministry of Health and population—67% Private healthcare providers—26% Non-governmental organisations/faith based Organisations/others—3%	By funding^c^: Public sector—23.28% Private sector—70.30% Other—6.42%
Form of decentralisation	Decentralised to district level	Federalisation and decentralised to local government/municipality	Devolution to state level (18 states)

Data sources: ^a^ National health Workforce Accounts [18]

^b^Malawi HRH strategy 2018 [19]

^c^System of health accounts report [Sudan] [20]

^d^NHWA Portal https://apps.who.int/nhwaportal/Home/Index

### Document review

The review examined the normative guidance on the areas of (a) HRH stakeholder coordination and (b) management through HRH units (or equivalent structures) within a governance framework globally as well as in the three study countries.

At global level, we searched for: journal articles through MEDLINE and Google Scholar and for policy documents from organisations and websites from 2004—the start of major HRH strengthening interventions with the launch of the ‘Joint Learning Initiative (JLI) report’ [21].

At country level, we searched for relevant journal articles through MEDLINE and Google Scholar and for policy documents. We followed up references of relevant documents and on advice of key informants. Table 5 provides details on the numbers and types of documents reviewed.

**Table 5:** Number and type of documents reviewed

Location	No. of documents reviewed	Type of documents reviewed
Global	95	Journal articles; documents from WHO HQ and regional offices, the Global Health Workforce Network and its predecessor the Global Health Workforce Alliance, regional HRH organisations such as the Asia Pacific Action Alliance on Human Resources for Health (AAAH), contemporary global HRH projects such as CapacityPlus and HRH2030; websites of international organisations (such as WHO and the World Bank)
Malawi	19	Relevant journal publications; current and recent HRH policies, strategies and reviews; wider contemporary policies, strategies and reviews on HRH and other factors impacting on HRH governance, coordination and leadership and management such as budget reform, civil service reform and decentralisation; HRH project reports; websites of international organisations (such as WHO and the World Bank), those working in the health workforce field and ministries of health, professional bodies and development partners in each of the study countries
Nepal	33	
Sudan	18	
Total	165	

We screened each title and abstract and where it was identified as being relevant, we read the full document and included it in the final analysis. We identified 165 documents as shown in Table 5.

### Key informant interviews

Using country and respondent-tailored topic guides, interviews carried out between July and September 2021 explored the functions of HRH Coordination Mechanisms and HRH Units, key attributes related to HRH governance, including participation, accountability, organisational adequacy, sustainability, and HRH performance/outcomes. The key informants were purposively selected based on: their experience of working or advising at strategic level on HRH coordination, leadership and management in the government health sector; knowledge of contemporary HRH situation at national and sub-national levels including policies, systems and practices. This enabled a mix of perspectives which supported our approach of triangulation.

Table 6 provides an overview of the key informants. The research team conducted the interviews virtually via Zoom or Microsoft Teams, in English and they lasted between 60 and 90 min. They were recorded following consent of the participants.

**Table 6:** Overview of key informants

Study site	Key informants	Number (female)
Global (GLO)	HRH experts at global or regional level working in international organisations or as consultants	7 (2)
Malawi (MWI)	National HRH stakeholders, including government officials at national and sub-national levels, development partners/donors, regulatory bodies, UN agencies	6 (0)
Nepal (NPL)		6 (1)
Sudan (SDN)		8 (5)
Grand total		27 (8)

### Data management and analysis

We transcribed verbatim the recordings of the interviews. For data anonymisation, we provided a site code (see Table 6) and serial number for each transcript, e.g. (GLO 001, SDN 004). We used the thematic framework approach to analyse the interview and document review data, supported by NVivo programme [22]. We developed a coding framework for all settings from the topic guides, research objectives and themes emerging from reading the transcripts and desk review information whilst being informed by dimensions of the HRH governance framework. We applied the framework to the transcripts and data and developed charts for each code. We then identified and agreed key themes.

## Results

### HRH coordination mechanisms

This section reports on the coordination mechanisms, their main functions and selected attributes (particularly the wider coordination mechanisms) including leadership and accountability; participation, inclusivity and consensus building; sustainability; and finally, performance.

#### Coordination mechanisms

Multiple coordination mechanisms were identified in all three study countries (see Additional file 1: Table S1). Malawi’s HRH TWG and Sudan’s National Stakeholder Forum have broad stakeholder reach and appeared to be long standing and embedded in the MoH systems. Nepal does not have a fully institutionalised health workforce stakeholder coordination mechanism. However, it has general coordination mechanisms that include health workforce and has ad hoc task-focused mechanisms such as the HRH roadmap working group.

#### Attributes

The important attributes of the coordination mechanisms cover leadership and accountability; participation, inclusivity and consensus building; and sustainability.

##### Leadership and accountability

The TWG in Malawi is led by the MoH and is accountable to the broader Health Sector Working Group and the MoH Senior Management Team, *“who decides whether to allocate funds to execute” (*MWI 001). The National HRH Committee in Sudan is also led by the Undersecretary for Health and accountable to the President, which gives it power. The interdivisional coordination mechanisms in the MoHP in Nepal report to the Secretary in the MOHP, and the HRH Roadmap TWG was led by the joint-secretary and reported to the Secretary MoHP.

The coordination mechanisms in Sudan had clear Terms of Reference (TOR) and guidance documents which describe the roles and responsibilities of the members, the reporting system, and schedule of meetings, with the HR observatory acting as the secretariat. In Malawi, the HRH TWG and the task forces reportedly had clear TORs. There are clear procedures for MoHP interdivisional meetings in Nepal. These instruments, along with the leadership capabilities of senior management (GLO 001), enhance the legitimacy of the mechanisms.

Leadership capacity was impacted by high staff turnover in Malawi and Sudan (partly through migration). HRH coordination mechanisms should address national rather than donor-driven priorities (GLO 003), but because of frequent changes in leadership in the MoHP (NPL 002, NPL 003 and NPL 005) the TWG in Nepal was driven by a development partner. A participant in Malawi referring to the development of the HRH Strategy described how development partners ‘*jumped in’,* ‘*took the heavy lift’*, and how a “*team of experts really pushed with the HRH Directorate, and then it came to reality*’(MWI 006).

The health workforce leadership needs to continuously engage and initiate dialogue with the Ministry of Finance (MoF) so that there are funds behind the priorities and actions identified [12]. Some respondents identified that these actors are often ‘not at the table until a later stage”, at which time they are reluctant to invest in health or the HW (GLO 007; GLO 006). Others have suggested that even when MoF is involved in these HRH coordination mechanisms, macro-economic and investments decisions are often made outside these mechanisms. Limited fiscal space, and international and domestic pressures to comply with the ‘austerity agenda’ and maintain ‘fiscal stability’ can also sway decisions to make long-term investments in the HW (GLO 006).

##### Participation, inclusivity and consensus building

The Malawi TWG had wide participation (see Additional file 1: Table S1). In Sudan, the Stakeholder Forum had a similarly wide reach of stakeholders with additional attendance by police and military HRH representatives. Respondents highlighted the challenge of leading and maintaining engagement of such large and diverse groups; this required effective communication and trust building efforts. In Nepal and Malawi, the composition of the working groups depends on the task, but donors and development partners will usually participate if they are contributing funds. In Malawi, participation by some MoH Directorates can be sporadic. At one point Sudan had dedicated staff and budgets to promote stakeholder engagement. In all three cases, monetary incentives were needed to encourage participation.

In Sudan, development partners, including UN agencies, have their own forum for general coordination which is represented in the NHC. In Malawi, development partners are members of the TWG, and one is always the co-chair. In Nepal the composition of the working groups depends on the function, but development partners contributing funds usually participate.

There is rapid expansion of private health worker training in Nepal and Sudan. The for-profit private (health) sector, which lack representative bodies, was absent from the HRH coordination mechanisms. Moreover, in Sudan, the private (health) sector remains unconvinced of the value in sharing data or the benefits of participation. Civil society was also missing from HRH coordination in the study countries.

Tensions and conflicts between stakeholders were reported due to the politicisation of health and healthcare funding and the presence of many powerful actors who believe they have a legitimate HRH governance and gatekeeping remit (GLO 002; GLO 006). These tensions were often intersectoral, such as education and defence (e.g. reluctance of security forces to share information in Sudan) or as a result of political transitions and resultant changes in institutional roles and responsibilities (GLO 001 and GLO 008). However, consensus-building, collective agenda setting, sharing objectives, use of workshops and informal communication helped to foster collective insight and views on the HRH topic, which eased tensions. Several global respondents observed that this process also improved HRH literacy amongst stakeholders and in Sudan *‘…the structures for coordination and the meetings, the culture of frequent meetings, has done a lot to mediate this relationship and to address conflicts.*” (SDN001).

##### Sustainability

Many respondents emphasised the need for a sustained forum for developing and overseeing the long-term strategy for the health workforce. The longevity of the HRH TWG in Malawi, despite high turnover of government staff, was attributed in part to its embeddedness within existing MoH governance structures and the perception by stakeholders that it was “*a competent structure”* and essential to the coordination of new initiatives: *“a donor wouldn’t really commit into a serious undertaking before being convinced that the TWG has reviewed and is happy with the direction”* (MWI 005).

In contrast, the functioning of the high-profile Stakeholder Forum in Sudan was affected by the recent political transition in the country. One respondent claimed that “*it* [Stakeholder Forum] *was functioning, though the country was unstable politically and the issues around the revolution make it a bit difficult to have the regular meetings as it is scheduled in the plan.*” (SDN 004).

Holding regular face-to-face meetings can be expensive if held in hotels and travel costs are required, though for smaller meetings one respondent (NPL 005) said that paying for a “few cups of tea” was a good investment if it helped bring people together. In Sudan, the Stakeholder Forum had dedicated government funding and commitment, with the majority provided through external partners and donors, though “*sometimes* [per diems] *it’s equal* […] *maybe to their* […] *monthly salary. So this is one of the things that really motivate people to attend”* (SDN 009). During the pandemic in Malawi, the opportunity to hold virtual meetings meant more people were available, meetings were more frequent and cheaper.

##### Performance

The coordinating mechanisms in Sudan and Malawi and the Road Map working group in Nepal had all supported the development of HRH strategic plans to support the long-term health workforce strategy. The coordinating mechanisms in Sudan and Malawi appeared to meet regularly, though this became difficult in Sudan after the 2019 revolution. Effective HRH coordination mechanisms were reported in Indonesia under the UHC umbrella which shared information and planning processes (GLO 006); and in Mozambique where a strong champion created the coordination mechanism which was supported by an HRH observatory. They gained the interest of stakeholders by demonstrating at the health system annual reviews that “*even if the issue is not a workforce issue, if you bring it their attention, and workforce component will be looked at*” (GLO 003). Two global respondents compared the challenge of coordinating multiple stakeholders in larger—especially federated—countries with smaller countries where all stakeholders could be “in one room” (GLO 002).

### HRH units

This section reports on findings about the structures of HRH Units, their functions and attributes (leadership/accountability, capacity, support to decentralised HRH units), and performance.

#### Types and functions of HRH units

All 11 countries in the Southeast Asia region reported in 2019 that they have some form of health workforce unit, compared with eight in 2018 [23], though in Timor-Leste “*it was just a one-person show.*” (GLO 001). In the African region, 15 out of 16 countries surveyed: “*had a responsible HR unit … but in practice, what had happened was that it was not really functional, many of them were just passing papers in practice*” (GLO 003). Below we have listed selected findings relating to the functions of the HRH units (or equivalent). Additional file 1: Table S2 shows that whereas Malawi and Sudan have clear HRH units to oversee health workforce functions, in Nepal there was no single structure to provide this oversight.

##### HRH strategy: development and implementation

HRH functions need to be coordinated within the MoH (GLO 008). A HRH strategic plan is needed for both stakeholders and within the MoH to guide, coordinate, and align HRH initiatives to longer-term health sector plans and fiscal and budgetary space to ensure long-term investment in the HW [12]. Nevertheless, the findings showed that Malawi was the only study country with a costed plan (2018–2022) currently being implemented, although there was no evidence that “costed annual implementation plans” ([19], p93) proposed in the plan have been developed or approved and/or funds allocated for activities not budgeted in the health sector strategic plan. The development of the Nepal HRH plan appeared to be very time consuming, often getting stuck at the approval stage with Nepal’s 2020–2030 plan only recently signed off. As a pathfinder country for GHWA, there was some external funding for developing Sudan’s 2012–2016 HRH strategy, but the process was apparently owned by the MoH and national stakeholders. In contrast, according to some respondents, the development of the HRH strategy in Malawi and Nepal was strongly influenced by development partners. Investment and therefore, implementation may be hampered without alignment to the fiscal and budgetary space. Although Nepal’s 2011 HRH strategy was officially approved and aligned to the health budget the funds to implement the planned activities were “frozen” ([24], p41). Dissatisfaction with the financing and implementation of Malawi’s current strategy was expressed: “*you need to have a proper budget, you need to have a proper plan, indicators, whether you meet those things or not, so, there should be that kind of platform”* (MWI 006)*.*

One global respondent remarked that in many countries HRH departments do not operate at a strategic level and are mainly focused on routine recruitment and deployment (GLO 001). Sometimes major HRH changes may be taken on by a different department. The ‘employee adjustment process’ to support federalisation in Nepal was not managed by HRH officials, but a focal person of the rank of Chief Specialist was appointed to manage this process (NPL 002).

##### Workforce planning and HR information

Though workforce planning is often a “*self-contained exercise within the health sector carried out in relative isolation from other development processes”* ([25], p359), in Malawi the staffing projections were part of the wider strategic HRH plan. The intelligent usage of HRH data [26] is needed for workforce planning and other workforce management processes. WHO has supported Health workforce observatories to generate such data. In 2015, 34 member states in the AFRO region had these observatories—including Sudan [27], yet most countries struggle to get accurate, comprehensive and current data on the workforce and only nine are currently active in the African region [28]. Despite years of donor support, the dedicated HRH information system in Nepal had failed and reliance of the personnel information system (PIS) for civil servants—including health workers—is only of “*some limited use for the training and other planning purposes*” (NPL 002). Sudan’s donor support to HRH information systems was curtailed by political sanctions. In Malawi, several information systems were in place, but the outputs could not be combined to produce useful information. HRH data sharing is limited in many countries, both within the Ministry of Health, itself and between ministries, such as Finance and Labour; “*they don’t talk to each other at all*” (GLO 001). However, in Indonesia, HRH data sharing between ministries was spelled out in a memorandum of understanding “*being very clear what data is going to be shared, when, by whom, and which platforms and everything … they were very systematic on that*” (GLO 006).

##### Lessons from COVID-19 about existing functions

A report from the South East Asian region suggested that lessons from COVID-19 on surge management and protection of health workers should be integrated into updated national HRH strategies [29]. Respondents described how COVID-19 “*exposed and amplified country weaknesses around “numbers of staff, distribution, skills*” (GLO 002; GLO 007), as well as data on the impact of the pandemic on the HW, for example absence due to medical and non-medical causes. It demonstrated clearly the importance of valuing, protecting and investing in the HW (GLO 001), [30]. One study highlighted how little evidence was available to healthcare managers and decision-makers in developing workforce strategies to respond to COVID-19 [31]. Dramatic changes in HW policy, regulation, legislation were observed as well as “emergency investments” in HW ‘surge recruitment’, and a flexibility in governance and financing mechanisms that seemed impossible previously, all of which allowed a more effective health workforce response to COVID-19 (GLO 001; GLO 002:GLO 007), [32]. Some respondents observed that some decisions were politically motivated, with governments keen to demonstrate they were doing something (GLO 007). In Malawi, in response to the COVID-19 pandemic and to avail of funding from the Global Fund, recruitment processes that normally take six months were completed “within two weeks or even less than that, and that is without compromising any quality” (MWI 006). While some respondents wondered whether things would default back as and when things get better, some were hopeful that with the improved understanding of HW complexities and the need for coordinated policy responses as a result of the pandemic, these levels of flexibility and responsiveness could be sustained (GLO 001; GLO 002).

##### Missing HRH functions

In Sudan the HR manual identifies the need for an employee relations unit, and this has been recommended in Nepal [33]. However, in spite of the risk of industrial action generally [28] and in all study countries some of which was related to COVID-19, there was no evidence of the practice of ‘employee relations’ within MoH structures.

#### Attributes

Three important attributes of the HRH Units emerged from the findings: leadership and accountability; capacity of HR unit staff; and support to decentralised units.

##### Leadership and accountability

The success of Sudan’s HRH Directorate was attributed to the leadership’s clear vision and ability to think “*outside the box and how to conduct things not like … routine”* (SDN 009). Elsewhere, HRH units may be hampered by unclear mandates and weak coordinating powers [8] or be positioned low in the organisational hierarchy excluding them from strategic decisions-making [34]. Leadership at levels above the HRH unit was also cited as being important to the creation and functioning of such a unit. Strong support was demonstrated in Sudan, but despite numerous calls for its establishment the Personnel Administration Section in Nepal has not been replaced by dedicated HRH Division—“*this is the leadership matter*” (NPL 002). Weak leadership at both levels will affect accountability. Lack of ownership where initiatives were driven externally, such as the development of strategic HRH plans, was also found to be associated with lack of accountability.

##### Capacity of HR unit staff

The global HRH strategy [3] promotes the need for a professionalised body of HRH scientists and planners and as well as policy-makers who understand and can support HRH at a strategic level. Some respondents reported HRH expertise in selected high-income countries, but a recent study in the South-East Asia region found that only 14% of staff in the HRH units were professionals (e.g. with Master’s Degree in Public Health) [23] and in the African region only 7% of staff were described as ‘technical’ [28]. In Malawi and Nepal, the HRH functions are staffed by people from “common services” ministries with knowledge of routine personnel administration, but who may be unfamiliar with the complexities of developing and managing a health workforce. One respondent said the perception is the management of public health staff and physicians or nurses was the same as managing agriculture staff, for example. *“They look* [at] *everything as … general*.” (NPL 006). A respondent from Malawi observed that generally “*people who are thrown to the HR department are those who are incapable, or who has a disciplinary issue or who want to have some calm time, so that they will do their own things. So, that is the debacle and because of those things always you see capacity issues”* (MWI 006). Another respondent suggested that in order to have a “*mature discussion around the intersectoral nature of the health workforce agenda*” it is critical to have policy-makers with ‘literacy’ in health workforce, e.g. able to think about “’*terms and conditions of employment’, ‘productivity’ [and] ‘performance’ [and] ‘labour rights*’” (GLO 007).

Becoming ‘literate’ about the health workforce also takes time. One respondent at a senior level in Sudan had been working for many years in HRH. However, just when the officer can develop effective and appropriate strategies, they may be transferred: “*when they start to pick up things, they also move to the other institution. So that's really quite a big handicap for an institution as specialized as health*.” (MWI 003). A review from Nepal in 2013 showed very high turnover of staff working on HR functions, especially those in leadership roles [33]. High turnover of senior managers has continued in Nepal, some of which is associated with political instability. In Sudan, training in health workforce development contributed to improved capacity of the HRH directorates at State and National levels. In the absence of a stable body of HRH professionals, some countries have relied on the use of international consultants with the risk that no expertise remains when the contracts finish [8]. *“In Burkina* [Faso] *they have received support from I think Belgium cooperation to have again an expert to strengthen the HRH unit. And they have done a good job to strengthen HRH information system at the national level. And then when the expat left, nobody was able to manage the system. Then the system died.”* (GL 004).

##### Support to decentralised units

Many countries either have or are moving towards decentralised health systems and management of the health workforce, as in the three study countries. This requires provision of support, including capacity strengthening, to the decentralised HRH units [3, 35]. Support strategies were included in Sudan strategic HRH plan for 2012–2016 [36] and implemented. One respondent reported that all 18 state-level HRH units (staffed with one or more focal persons) are functioning. Strategies to support decentralised HRH units in the federal, provincial, municipal structures were included in Nepal’s HRH Roadmap. Similar support to Malawi’s devolution was anticipated in its HRH strategic plan—the “d*evolution of the HR function led to a delineation of roles and responsibilities between the line Ministry (MoHP) and the Councils”* ([19, p16), but at the time of this study, institutional HRH roles and responsibilities, e.g. of the health service commission and the local government service commission at central and subnational levels had not been fully delineated.

#### Factors impacting on performance

A range of factors were found to impact on the performance of HRH Units, including: their legitimacy and power linked to positioning within the MoH structure and hierarchy (GLO 001) [10]; their political capital and engagement of stakeholders at the highest level, “*that gives you the power in order to bring different departments on the table”* (GLO 001); “*funding power”* (GLO 002) and their “capacity and policy space to plan, manage, cost and follow up all actors adhering to one HW plan” [12]. To maintain technical autonomy and financial and programmatic independence, availability and use of monitoring and evaluation instruments and HR data are needed to monitor, report on and be accountable for results. This requires the availability and retention of HR literate professionals [11].

## Discussion

### Key considerations in health workforce development for UHC

Building and maintaining a health workforce fit to support UHC requires the development and sustaining of a long-term HW planning and financing agenda informed by labour market analysis. The challenge is to facilitate and maintain a well-informed dialogue with a sufficient range of stakeholders [37, 38]; maintaining a focus on (investing in) the health workforce development to support long-term health plans, whilst appreciating the shorter-term needs of some HRH stakeholders; and whilst navigating an ever-changing environment (a range of which was demonstrated in the study countries) such as decentralisation, pandemics, health worker migration and political transitions, civil unrest [39]. Key to this process are:

- The leadership that recognises the health workforce as essential to achieving UHC and the need for a financial plan for long-term investment in the HW.
- Robust and transparent mechanisms that promote political and social accountability and responsibility for performance and results/health workforce outcomes.
- Well-informed stakeholders and decision-makers through provision of good HW information and building the kind of ‘health workforce literacy’ that enables them to plan for and understand changing health workforce dynamics.
- A unified approach to coordination of stakeholders who support the timely development and oversight of an appropriate and costed HRH strategy subsequently implemented and monitored [40] by an HRH unit (or equivalent organising structure).
- Adaptable and flexible HW governance arrangements and policies that can respond to shocks and emergencies.
- Reliable, timely and comprehensive HW data available at all levels to inform evidence-based decision-making.

### HRH coordination mechanisms

The study identified numerous coordination mechanisms which may be fit for their specific purposes, but to support the HRH long-term agenda they need to be sustained and inclusive of a wide range of stakeholder interests [41] including those of the private sector and civil society. Task-oriented groups can be helpful, but they should be integrated into the wider coordination mechanism. The coordination mechanisms need to be seen as credible, so stakeholders perceive a benefit or value in participation. Credibility is enhanced by organisational support and the generation and availability of timely HRH data. This can be achieved by various means, such as Sudan’s HRH observatory, though there are currently few functioning observatories in the African region. There is a need for intersectoral coordination mechanisms to be owned and driven by the MoH and not reliant on external funding as this jeopardises sustainability.

#### HRH units

We found no evidence that a single HRH unit is essential, but there should be a structure to provide the necessary technical leadership and coordinate the development of HRH strategy and the implementation of HRH activities. This is more complex, as in all study countries, when the management of health sector employees is spread across several government departments [42]. The absence of a distinct HRH unit may explain the leadership of the employee adjustment process in Nepal by another department in the MoHP, but even well-established HRH units may be side-lined regarding important HRH initiatives [43]. Coordination of HRH activities becomes yet more complex with the introduction of decentralised management structures. The speed of decentralisation may be unpredictable [35], but in Sudan this appeared to have been adequately planned for. There is a serious absence of expertise specific to managing the complexities of the health workforce. Surveys in Africa and South-East Asia report on small numbers of ‘technical’ or ‘professional’ staff [11, 28], but it is unclear if they have HRH expertise. However, much of this specialised knowledge—or ‘HRH literacy’—can only really be learned on the job. The high level of turnover in HRH units makes this difficult in many countries that second ‘common services’ staff to HRH positions, whose tenure is short-lived. Filling theses skills gaps with time-limited consultants does not build the capacity needed [8]. A major input for HRH management is data, especially for workforce planning and monitoring and evaluation. Much donor support has been provided to develop stand-alone information system, but this is often unsustainable. Further investigation of the Indonesian example of data sharing would be useful.

### Future actions

This study has revealed many of the complexities of HRH governance, but more information is needed. The COVID-19 pandemic has highlighted the need for more adaptable HRH governance arrangements which merit further exploration. One approach could be policy-makers to work with research groups to document—guided by areas covered by this paper—(a) the current state of HRH coordination mechanisms and their effectiveness, and (b) the structures and processes for managing HR functions and their effectiveness. Mapping the HR functions would help with the latter activity. An effective and costed HRH strategy based on a long-term vision that is actually implemented could result from these actions. The global HRH strategy [3] provides guidance on appropriate actions to strengthen these areas of HRH governance. This activity, in turn, would strengthen ‘health workforce literacy’ amongst stakeholders at all levels. With such a locally owned approach in place, Ministries of Finance and international donors could be approached to provide financial and technical assistance as needed. Documentation of this whole process would facilitate learning and benefit other countries.

### Strengths and limitations of the study

As intended, this study has investigated HRH coordination and HRH units in greater depth than the NHWA reporting and the more detailed surveys from several WHO regions [10, 11, 28]. Since signing up to the Kampala Declaration in 2008, all three countries have seen major changes and particular political transition and civil unrest in Nepal and Sudan which impacts on HRH governance. However, from our interviews with the global respondents and review of the literature, we feel that our general findings are quite representative of many other countries that signed the Kampala declaration and even some OECD countries. In addition, the study has identified important tangential issues—such as the HRH strategy and the interdependence of HRH coordination mechanisms and HRH units—which are central to effective HRH governance. Nevertheless, there were limits on the time-frame, the number of interviews (which had to be carried out online) and available country-specific documentation. These challenges resulted in some loss of detail—especially the nuances of the politics of HRH—and limited the degree of triangulation possible.

## Conclusions

The global HRH strategy provides a useful stimulus to strengthen HRH governance through mechanisms and actions to coordinate an intersectoral HRH agenda and structures to develop and monitor HRH policies and plans. Reporting on these activities through the NHWA encourages implementation and monitoring against the two milestones concerned at all levels. However, deeper understanding of the ways these mechanisms and structures operate is essential for enhancing their contribution to HRH governance and ultimately the HW contribution of achieving health goals. This paper, which provides some lessons, takes a first step in this direction.

## Supplementary Information

**Additional file 1: Tables S1 and S2.** Shows that whereas Malawi and Sudan have clear HRH units to oversee health workforce functions, in Nepal there was no single structure to provide this oversight.

## Data Availability

The datasets generated and/or analysed during the current study are not publicly available due confidentiality and ethical restrictions, but are available from the corresponding author on reasonable request.

## Abbreviations

AFRO: African Regional Office (WHO)
DHRMD: Directorate of human resource management and development (MoH, Malawi)
DHRMD: Department of human resource management and development (OPC, Malawi)
DoHS: Department of Health Services (Nepal)
EMRO: Eastern Mediterranean Regional Office (WHO)
FMoH: Federal Ministry of Health (Sudan)
GLO: Global (Respondent)
HRH: Human resources for health
HRM: Human resources management
HW: Health Workforce
LSTM: Liverpool School of Tropical Medicine
MoH: Ministry of Health (Malawi)
MoHP: Ministry for Health and Population (Nepal)
MWI: Malawi (Respondent)
NCHC: National Council for Healthcare Coordination (Sudan)
NHC: National HRH Committee (Sudan)
NHRHO: National Human Resources for Health Observatory (Sudan)
NHWA: National Health Workforce Accounts
NPL: Nepal (Respondent)
SEARO: South-East Asian Regional Office (WHO)
SDN: Sudan (Respondent)
ToR: Terms of Reference
TWG: Technical Working Group
UHC: Universal Health Coverage
WHO: World Health Organization
