# Supplementary material for: Improving health workforce governance: the role of multi-stakeholder coordination mechanisms and human resources for health units in ministries of health
Source: Hum Resour Health. 2022 May 26;20:47. doi: 10.1186/s12960-022-00742-z (PMC9134719; doi:10.1186/s12960-022-00742-z)
Supplement: Supplementary file 1 — Additional file 1: Tables S1 and S2. Shows that whereas Malawi and Sudan have clear HRH units to oversee health workforce functions, in Nepal there was no single structure to provide this oversight. [file 12960_2022_742_MOESM1_ESM.docx]

**Additional file 1**

**Table S1: Summary of coordination mechanisms and functions in study countries**

| **Country** | **Type of mechanism** | Summary of functions |
| --- | --- | --- |
| **Malawi** | Health Sector Working Group, MoH | - Oversees all MoH Technical Working Groups (TWGs), including HRH TWG - *The ultimate governance body for the health sector*; *the highest level governance body that can tackle HR issues when necessary* (MWI 003) - Comprises the Secretary for Health, donors, civil society, the private sector, other government departments, but *attendance of other Ministries, this also is something that's not really very strong* (MWI 003) |
|  | HRH TWG, under the Health Sector Working Group, MoH | - Multisectoral and multi-partner mechanism, established in the early 2000s; comprises approx. 30 members (covering multiple central government departments, local government, regulatory bodies, training institutions, the faith-based organisations, development partners and donors, some of whom provide technical and financial support to HRH functions) - Provides technical advice and generates evidence for MoH Senior Management Team HRH decision-making; *reviews and endorses national HR policies and plans* [19, p23] - *Monitors and reviews implementation of HRH Strategic Plan* against annual targets and planned activities [19, p17] - *Advocates for adequate funding* [19, p23] - Functions according to TOR, led by the MoH HR Director (Chair), with a development partner as rotational co-Chair, with MoH Directorate of Human Resource Management and Development (DHRMD) providing the Secretariat - Meets quarterly - A *think tank* that considers issues government cannot make a decision about (MW 001) |
|  | Task Forces, under the HRH TWG, MoH | - Constituted by the HRH TWG to work on specific HRH issues and tasks as per agreed ToR, e.g. the development of the HRH strategy, recruitment - Comprises volunteer TWG members and consultant hired when required - Mechanism *through which the TWGs deliver* (MWI 001) |
|  | Parliamentary Committee for Health | - Advocates for health including health workforce issues |
|  | Permanent Secretaries (PS) Committee | - Multi sectoral committee addressing health sector issues, includes Permanent Secretary for Health and for DHRMD in the Office of the President and Cabinet. among others - Health sector coordination and policy decision-making, and potential to influence HRH decision-making |
|  | MoH Senior Management Team | - Main decision-making body in the MoH, considers evidence presented by all TWGs, including HRH TWG for HRH decision-making and allocating resources |
|  | Human Resources for Health (HRH)Coalition | - Umbrella body for professional associations and unions including medical and nurses and midwives’ associations - Petitions government on various HRH issues on behalf of its members |
| **Sudan** | National Human Resources for Health Observatory (NHRHO) | - Established 2007 - Produces evidence and documents for review - Develops meeting agendas, follows up actions identified by the Stakeholders Forum |
|  | National HRH Committee held the National HRH Stakeholder Forum reporting to the National Council for Healthcare Coordination (NCHC) | - Multisectoral body - Headed by the Undersecretary for Health with links to the President and has “*the strength and the power and the authorization even to push HRH issues and agenda*” (SDN 004) - Serves as the Board for the NHRHO - Coordination and decision-making role, e.g. salary increases, distribution, production and migration, “*when the decision is taken in this committee, so it is likely to be done*” (SDN 002) - Meets quarterly |
| **Nepal** | Health Coordination Division, MoHP | - General health coordination role across the three tiers of government, including for HRH - Coordination with multisector including development partners |
|  | Ad hoc TWGs/committees, MoHP (set up for specific tasks) | - CCF process to develop 2011 HRH plan which included task-specific working groups - Development of the HRH Strategic Plan/Roadmap (2017-2020) co-led by MoHP and WHO - Committee for planning for the (re) deployment of staff - the Employee Adjustment process - under federalisation - Groups may comprise MoH officials and development partners |
|  | Interdivisional meetings, MoHP | - Weekly meetings to discuss policy issues, including HRH, but "*lack of clear agenda, preparation and participation*"(NPL 004). |

**Table S2: Summary of HRH units**

| **Country** | **Type of HRH unit** | **Description/function** |
| --- | --- | --- |
| **Malawi** | Directorate of Human Resource Management and Development (DHRMD), Ministry of Health (MoH) | Provides strategic direction on the rational use of HRH [19, pp23-4]  Headed by a Director who reports to the Principal Secretary for Health  Provides the secretariat for the HRH TWG  Leads the implementation of the HRH Strategic Plan (2018-2022)  Relies on external technical assistance to perform core functions such as workforce planning  Comprises health workforce planning; management; and development sections  The health workforce management section is responsible for a broad range of HRM functions, including the “*interpretation of the Malawi Public Service Regulations (MPSR) for central hospitals*” [19]  Works with the Health Services Commission, Local Government Service Commission, and District Councils which have responsibility for some HR functions |
| **Sudan** | Directorate General of HRH, Federal Ministry of Health (FMoH) | Evolved from personnel management unit in 2003 to a Directorate General of HRH Development, reporting directly to the Undersecretary for Health  Responsible for “*strategic health workforce development including the development of several policies for the health workforce and expanding its role in coordination and facilitation of the health workforce issues”* (SDN 001)  Includes 8 directorates and institutes to carry out functions with 150 staff: Policy and Planning, National HRH Observatory, Internship Affairs, Fellowships Affairs, Continuous Professional Development (CPD), Academy of Health Science (AHS), Public Health Institute (PHI) (now under the direct management of the Undersecretary of Health), Blue Nile National Institute for Communicable Diseases (BNNICD) managed collaboratively with the University of Gezira |
| **Nepal** | Personnel Administration Section, Division of Administration, Ministry of Health and Population (MoHP) | Covers all functions related with personnel administration, HRH information, communication and management  2012 Mid-Term Review of Nepal Health Sector Strategic Plan NHSP-2 recommended for “*a single human resources division and that all human resources activities are assigned to this division*” [24, p15] but not acted on. |
|  | Curative Service, Education, and Research Section, under Policy, Planning and Monitoring Division, MoHP | Responsible for coordination with health academia, education institutions, hospitals, and technical schools |
|  | Health Coordination Division, MoHP | Responsible for: the Employee Adjustment process; formulation of HRH policies and plans; workforce planning and projection; HR information systems; training needs assessment, training plans and career development; and HRH research [41]  Includes Provincial and Local Health Coordination Section  [41] |
|  | Administration Section, Department of Health Services (DoHS) | Personnel administration for staff employed by DoHS, including attendance and staff leave. |
|  | National Health Training Centre, Department of Health Services (DoHS) | Responsible for: HRH training strategy and plan; coordination for training and other capacity development initiatives for HRH; development and use of training materials for HRH |
|  | Nursing Capacity Development Section, under the Nursing and Social Security Division, DoHS | Coordination of planning, capacity development and management of nursing and midwifery work force |
